# Intra-annual Dynamics of Xylem Formation in *Liquidambar formosana* Subjected to Canopy and Understory N Addition

**DOI:** 10.3389/fpls.2018.00079

**Published:** 2018-02-05

**Authors:** Shaokang Zhang, Sergio Rossi, Jian-Guo Huang, Shaowei Jiang, Biyun Yu, Wei Zhang, Qing Ye

**Affiliations:** ^1^Key Laboratory of Vegetation Restoration and Management of Degraded Ecosystems, South China Botanical Garden, Chinese Academy of Sciences, Guangzhou, China; ^2^Guangdong Provincial Key Laboratory of Applied Botany, South China Botanical Garden, Chinese Academy of Sciences, Guangzhou, China; ^3^Département des Sciences Fondamentales, Université du Québec à Chicoutimi, Chicoutimi, QC, Canada; ^4^South China Botanical Garden, University of Chinese Academy of Sciences, Beijing, China

**Keywords:** microcoring, *Liquidambar formosana*, cambium, cell differentiation, growth, meristem, nitrogen, wood formation

## Abstract

Increasing N deposition caused by intensive anthropogenic activities is expected to affect forest growth. However, the effects of N deposition on trees are still controversial due to the wide variability in results and experimental methods used. We conducted an experiment involving both canopy and understory N addition to investigate the effects of N-addition on intra-annual xylem formation of Chinese sweetgum (*Liquidambar formosana*) in a warm-temperate forest of Central China. Since 2013, 50 kg N ha^-1^ year^-1^ (2.5 times the current natural N deposition) was applied monthly from April to December. In 2014 and 2015, the timing and dynamics of xylem formation were monitored weekly during March–December by microcoring the stems of control and treated trees. Similar dynamics of wood formation were observed between canopy and understory N addition. Xylem formation of all the experimental trees started in March and lasted for 119–292 days. Compared to the control, no change was observed in the timing and dynamics of wood formation in N-treated trees. Tree ring-width ranged between 1701 and 4774 μm, with a rate of xylem production of 10.52–26.64 μm day^-1^. The radial growth of trees was not modified by the treatments. Our findings suggest that short-term N addition is unable to affect the dynamics of xylem formation in Chinese sweetgum in Central China. The effects of N on tree growth observed in previous studies might be related to the duration of the experiment or the imbalance between the amount of natural deposition and N added during treatments.

## Introduction

Plant phenology, the timing and dynamics of plant development and growth, are physiological traits sensitive to the environmental changes (Schwartz et al., 2006; Cleland et al., 2007). In recent decades, anthropogenic emissions have induced profound changes in N availability worldwide (Allen et al., 2014). Increasing N depositions have greatly changed the cycle and availability of N for plants and are predicted to increase in the future (Galloway et al., 2004; Thomas et al., 2010). Tree growth, which is related with forest development and carbon cycle, is generally considered to be limited by N availability in the northern hemisphere (Aber, 2002; Fang et al., 2005; Zhao and Liu, 2009). Thus, understanding the effects of increased N deposition on tree growth can improve predictions of tree growth and carbon sequestration of forests for the future.

Concerns about the potential impacts of N deposition on trees and forest ecosystems have been increasingly addressed in recent decades (Bigras et al., 1996; Zhao and Liu, 2009; Bobbink et al., 2010; D’Orangeville et al., 2013; Plavcova et al., 2013; Dao et al., 2015). Based on the previous studies, diverging results may be expected. (i) N deposition would facilitate tree growth, generally in N-limited or young forests. In a warm-temperate ecosystem, *Picea aspirate* Mast. and *Pinus tabulaeformis* Carr. started growing earlier and produced more biomass after N fertilization (Zhao and Liu, 2009). (ii) Overloading of N inputs could lead to soil acidification with negative effects on tree growth (Katzensteiner et al., 1992; Bobbink et al., 2010). In a greenhouse experiment, Kula et al. (2012) found that 178–535 kg ha^-1^year^-1^ of N addition induced a decrease in height and diameter increment of *Betula pendula* Roth., associated with delayed bud break. (iii) No significant change was observed in tree growth after N addition (D’Orangeville et al., 2013; Lovett et al., 2013; Dao et al., 2015). Dao et al. (2015) found that a 6-year long N application did not increase the xylem production of *Picea mariana* Mill. in Canada. These diverging results represent a great challenge to understanding how tree growth will respond to the ongoing increasing N deposition.

Most experiments have applied N directly to understory and soil to simulate the effects of increasing N deposition on tree growth, thus ignoring the potential processes happening in the canopy (Gaige et al., 2007; Lu et al., 2010). In fact, most deposited atmospheric N is inevitably first intercepted by the tree canopy. Deposited N would thus be absorbed or adsorbed by leaves, epiphytes and microorganisms, retained on the bark, immobilized in decaying leaves, twigs or other dead organic matter in the canopy, or transformed from inorganic N to organic N (Cardelus et al., 2009; Dail et al., 2009; Sparks, 2009; Matson et al., 2014). In this case, these potential processes would change the quality and quantity of N that is available for tree growth (Houle et al., 2015). A study conducted in a subalpine forest found that approximately 80% of total N deposition during the growing season was retained by foliage and branches. This retention caused greater photosynthetic efficiency and higher carboxylation rates that raised production in spruce branches (Sievering et al., 2007). A fertilization applied from the top of the canopy would therefore be more appropriate to simulate the natural increasing N deposition than the understory N addition method (Zhang et al., 2015). However, to our knowledge, few studies have been conducted to compare the impact of N on wood formation between understory and canopy fertilization. Whether the understory treatment overestimated or underestimated the influences of the natural N deposition on radial growth of trees thus remains unclear.

The aim of this study was to investigate the effects of understory and canopy N addition on wood formation. From 2013 onward, we conducted an experiment in a warm-temperate forest in Central China, where 2.5 times the current natural N deposition of inorganic N was applied either to the canopy or understory of sweetgum *(Liquidambar formosana* Hance.). The growth of xylem production was monitored weekly during 2014–2015 to test the hypothesis that N addition may stimulate wood formation. Based on previous studies conducted at the same site, the understory N addition (2.5 times the current natural N deposition) was not able to affect soil chemistry (e.g., pH, N, K, Ca, Al) (Zhang et al., 2015; Shi et al., 2016). Besides, it is known that leaves are able to absorb the deposited N for growth before it reaches the soil (Munoz et al., 1993; Reich et al., 1998; Bondada et al., 2001; Sievering et al., 2007). Therefore, we aimed to answer the question whether canopy N addition is more effective than understory N addition in stimulating wood formation.

## Materials and Methods

### Study Site

The experiment was conducted in a natural mixed forest in the Jigongshan National Nature Reserve (31°51′ N, 114°05′ E, 300 m a.s.l.), Henan Province, Central China. The experimental site is located in the transitional zone between the subtropical and warm-temperate climate region dominated by sweetgum (*L. formosana*), the studied species. The stand is even-aged with 45-year-old trees growing in soil classified as yellow-brown loam (Zhang et al., 2015).

Based on 60-year climatic data^1^, mean annual temperature is 15.2°C, with the highest and lowest monthly mean temperature of 27.5°C and 1.9°C in July and January, respectively. Average annual precipitation is 1119 mm, of which 80% falls during April–October. On average, rain provides a natural deposition of 19.6 kg N ha^-1^ year^-1^ (Zhang et al., 2015), in which the ratio of NH_4_^+^:NO_3_^-^ is close to 1.

### Experimental Design and Tree Selection

A random design was used, in which four blocks were established for the N fertilization experiment. Three plots were randomly assigned within each block and subjected to different treatments: control (abbreviated as C), understory N addition (UN) and canopy N addition (CN). Each Block and plot have an area of 3,628 m^2^ and 907 m^2^, respectively. polyvinylchloride (PVC) board was installed and more than 20 m of buffer zone was set up between two adjacent plots to avoid contamination of N solution and interactive influence of tree growth between plots. Since the locally naturally deposited N is mainly consisted of NH_4_^+^ and NO_3_^-^ and the ratio between them is close to 1, ammonium nitrate was used in this experiment. The treatments consisted of sprinkling an ammonium nitrate solution as homogeneously as possible at 1.5 m above ground (UN) or from the top of the tree canopy (CN). The control remained untreated. In each plot, one healthy adult Chinese sweetgum with upright stem was selected. Overall, the analysis involved four C-trees, four CN-trees, and three UN-trees, as one UN-tree died in the second experimental year.

From 2013 onward, the equivalent of 3 mm of rain containing the N solution was applied to the treated plots once a month during the growing season (April–October), for a total deposition of 50 kg N ha^-1^ year^-1^. Overall, the treatment provided the trees with 21 mm of additional rain per year, representing < 1% of the total annual precipitation at the site. The confounding effect of water addition by the treatment is therefore considered marginal.

### Microcores Collection and Preparation

During 2014–2015, wood microcores were collected weekly from March to December following a spiral trajectory up the stem with a Trephor (Rossi et al., 2006a). This sampling method assured that the samples collected from around the stem could represent the radial growth of the tree. Wood microcores were taken at least 5 cm apart from one another to avoid disturbance by previous samplings. The microcores contained at least three recent tree rings and the developing annual layer with cambial zone and adjacent phloem tissues. They were immediately put in Eppendorf micro-tubes containing 50% aqueous ethanol solution and stored at 4°C.

A total of 726 microcores were immersed in ethanol and D-limonene solutions, and then embedded in paraffin (Rossi et al., 2006a). Transverse sections of 8–12 μm were cut from the samples with a rotary microtome. The sections were stained with cresyl violet acetate (0.05% in water), and observed under visible light at magnifications of 400× to differentiate the cambium and developing xylem (Rossi et al., 2006a). In cross section, cambial cells were characterized by thin cell walls and small radial diameters (Rossi et al., 2006b), while the radial developing xylem cells had at least twice the radial width of the cambial cells. The total width of differentiating and mature xylem was measured along three radial files on images captured at magnifications of 100× with a camera fixed on a microscope using LAS software (V4.6, Leica Microsystems, Germany).

In January–February 2016, 5–10 additional microcores per tree were collected and prepared using the abovementioned procedures. The width of the xylem produced during 2011–2015 was measured at magnifications of 50× along three radial rows and averaged to quantify the radial growth patterns before and after N treatments.

### Chemical Analysis of Soil and Leaf, and Leaf Size Measurement

Soil and leaf samples were collected for chemical analyses in August 2016. Five cylindrical soil cores (0–10 cm depth) were taken in each plot, and pooled together for chemical analysis. Leaves were gathered at different tree heights to be representative of the canopy. N content was measured by micro-Kjeldahl digestion and the indophenol blue method (Liu, 1996). Leaf size was measured on 10–20 leaves collected from each tree during October–November 2014 and 2015 using a leaf area meter (Yaxin-1241, Beijing Yaxin Science & Technology Co., China).

### Curve Fitting and Statistics

Chemical contents and leaf size were compared between treatments using analysis of variance (ANOVA). When the effects were significant, Tukey–Kramer tests were used for multiple comparisons. The tree-ring width was analyzed with repeated measures ANOVA to compare treatments including the year as a repeated factor.

Xylem growth data were fitted to Gompertz functions defined as:

$$y\,\;\;=\;\;A\,\;\exp\;[-e^{\left(\beta -kt\right)}]\,\;$$

where *y* represents the cumulative width of new produced xylem and *t* day of the year (Rossi et al., 2003). The three parameters are the upper asymptote *A*, the *x*-axis placement parameter *β* and *κ* the rate of change in shape. The residuals were regressed onto the partial derivatives with respect to the parameters until the estimates converged. Several possible starting values were specified for each parameter, so that the procedure evaluated each combination of initial values using the interactions and producing the smallest residual sums of squares.

Curve fitting was bootstrapped 10,000 times for each dataset, i.e., treatment and year. The 2.5 and 97.5% percentiles, which include 95% of the values, were extracted from the distributions of the three parameters of the Gompertz function. Significant differences between groups were considered at *p* < 0.05 when the confidence intervals from two distributions did not overlap (Chemick, 2008). Statistics were performed using SAS 9.4 (SAS Institute Inc., Cary, NC, United States).

## Results

### Chemical Analysis and Leaf Size

Total N in the soil varied between 2.01 and 2.33 g kg^-1^, with no significant difference detected among treatments. NO_3_^-^ ranged from 6.66 to 8.84 mg kg^-1^, with no difference among treatments. NH_4_^+^ varied between 6.35 and 16.5 mg kg^-1^, with higher content detected in UN, while no difference was observed between C and CN. Similar pH values of 4.14–4.31 were observed in all treatments. Total leaf N and leaf size varied between 13.54 and 20.48 g kg^-1^ and between 4581 and 5240 mm^2^, respectively, without differences detected among treatments (**Table 1**).

**Table 1:** Soil and leaf N content and leaf size in control (C), canopy N addition (CN) and understory N addition (UN) treatments in a warm-temperate forest, Central China.

	Treatment		
	C	CN	UN
Soil total N (g kg^-1^)	2.01 ± 0.49^a^	2.33 ± 0.37^a^	2.21 ± 0.12^a^
Soil NO3- (mg kg^-1^)	6.66 ± 1.83^a^	8.84 ± 2.43^a^	7.2 ± 2.31^a^
Soil NH4+ (mg kg^-1^)	6.35 ± 2.94^a^	7.43 ± 3.95^a^	16.5 ± 3.11^b^
Soil pH	4.31 ± 0.16^a^	4.21 ± 0.16^a^	4.14 ± 0.07^a^
Leaf total N (g kg^-1^)	13.54 ± 2.86^a^	20.48 ± 4.76^a^	16.31 ± 3.18^a^
Leaf size in 2014 (cm^2^)	45.81 ± 15.57^a^	50.34 ± 17.28^a^	50.43 ± 15.38^a^
Leaf size in 2015 (cm^2^)	52.41 ± 13.43^a^	46.63 ± 10.38^a^	46.93 ± 14.82^a^


*Values are reported as mean ± standard deviation. Different letters represent significant differences between treatments*.

### Intra-annual Xylem Production

**FIGURE 1:**
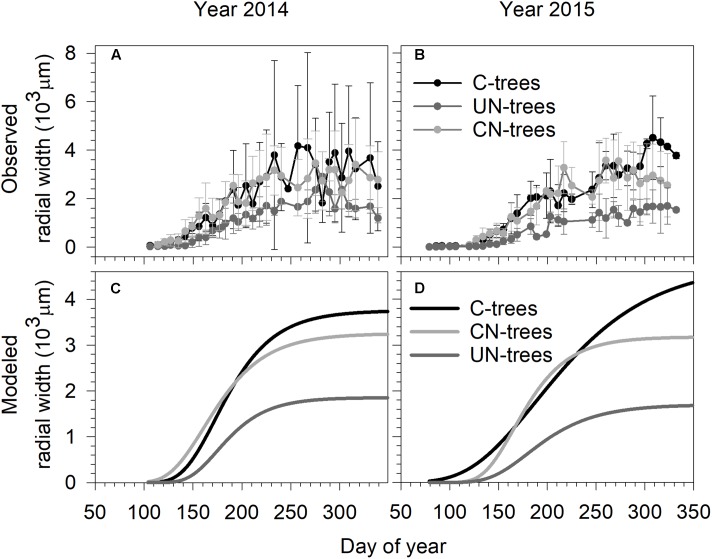
Observed **(A,B)** and Gompertz modeled radial growth **(C,D)** of sweetgum under control-C, canopy N addition-CN and understory N addition-UN treatments during the 2014 and 2015 growing seasons in Central China. Vertical bars represent the standard deviation among trees.

In 2014, xylem growth of all trees started before the first sampling on DOY 106 (mid-April) (**Figure 1**). In 2015, xylem formation started on DOY 86 (end of March) in C-trees and UN-trees, and on DOY 92 (beginning of April) in CN-trees (**Figure 1**). In both years, data were more dispersed in C-trees than CN-trees and UN-trees due to the presence of outliers, which were not removed from the dataset because of the reduced number of replicates.

**FIGURE 2:**
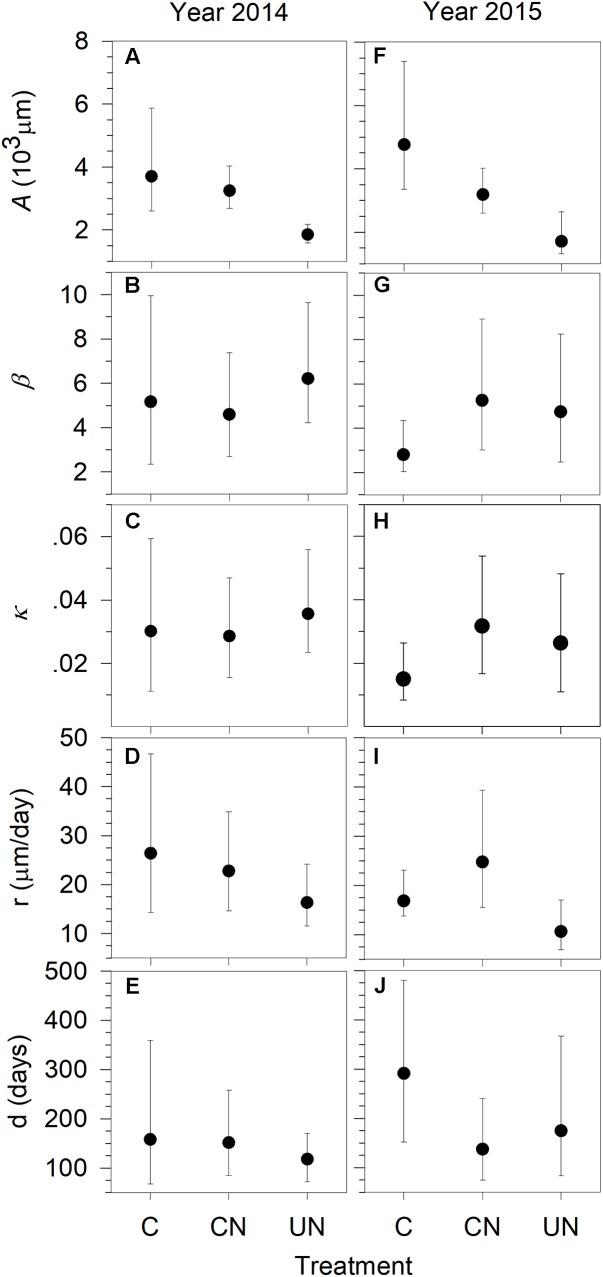
Parameters *A* **(A,F)**, *β* **(B,G)**, *κ* **(C,H)**, of the Gompertz functions fitting xylem production and *r* (growth rate) **(D,I)** and *d* (duration) **(E,J)** of sweetgum under control-C, canopy N addition-CN and understory N addition-UN treatments during 2014–2015 in Central China. The vertical bars represent 2.5 and 97.5% confidence intervals of the distributions calculated by 10,000 bootstrapped replications. Significant differences between groups were considered at *p* < 0.05 when the confidence intervals from two distributions did not overlap.

As indicated by the asymptote *A*, in general, trees produced xylem with a total width of 1701–4774 μm per year, with the significantly smaller xylem production (1701–1857 μm) in UN-trees (**Figure 2**). UN-trees had lower xylem production than C-trees and CN-trees in 2014, and lower than C-trees in 2015. The x-axis parameter (*b* in **Figure 2**) and the rate of change of the growth curves (*κ* in **Figure 2**) ranged from 2.80 to 6.14, and from 0.0149 to 0.0352 during 2014 and 2015, respectively. Xylem production rate (*r*) varied between 10.52 and 26.64 μm day^-1^, with the lowest observed in UN-trees (**Figure 2**). Duration of xylem production (*d*) ranged between 119 and 292 days, with C-trees showing the longest durations in both study years (**Figure 2**). Except for the asymptote, no significant difference in the other parameters was detected between treatments, indicating that all trees had similar patterns of xylem growth but different cell production.

### Inter-annual Xylem Production

**FIGURE 3:**
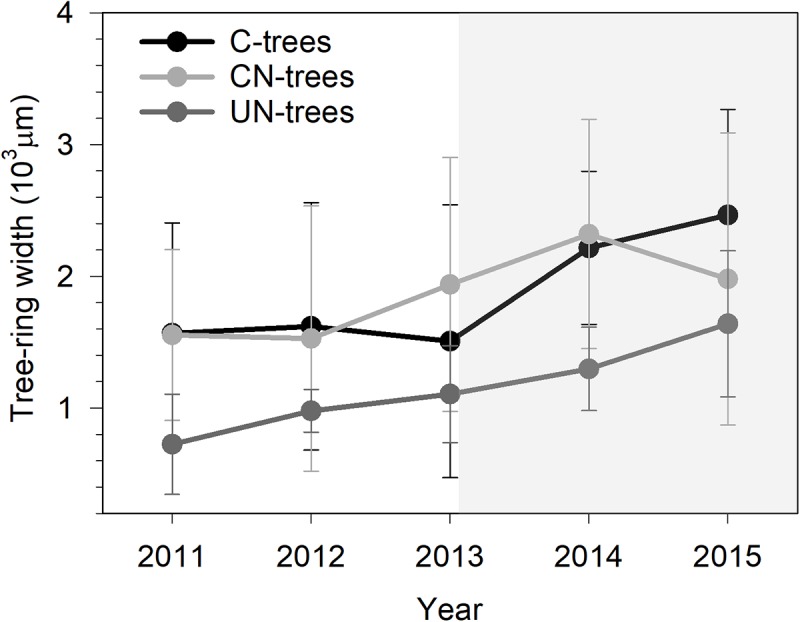
Tree ring-width of sweetgum in control-C, canopy N addition-CN and understory N addition-UN trees during 2011–2015 in Central China. Bars represent the standard deviation among trees. Gray background indicates the period of N addition.

Xylem production differed between years (**Table 2**, *P* < 0.0001), with the width ranging from 725 μm in 2011 to 2465 μm in 2015. UN-trees produced between 725 and 1639 μm xylem width, but was significantly lower than C-trees (1507–2465 μm) and CN-trees (1528–2320 μm) only in 2014 (**Figure 3**), which was consistent with the Gompertz predicted productions modeled based on the observed data. Although the variance between years was larger than that in treatments, and xylem production was significantly different between years and treatments (*P* < 0.05), no significant effect of treatment × year interaction was detected by repeated measures ANOVA (**Table 2**, *p* > 0.05), which indicated that the N addition was unable to modify the radial growth of trees.

**Table 2:** Comparison in the radial growth of sweetgum between control (C), canopy N addition (CN) and understory N addition (UN) treatments using repeated measures ANOVA in Central China during 2011–2015.

Source of variation	*F*-values	*P*
Treatment	7.81	0.02
Year	3.20	0.02
Treatment × year	0.75	0.64


## Discussion

In this study, N addition was applied either in the understory or above the canopy to test the effects of N deposition on stem xylem formation in a natural warm-temperate forest in Central China. Although the xylem production in UN-trees showed less than C-trees and CN-trees, this difference was already present before N addition. Based on the statistical analysis, no effect of the N addition was found on timing and dynamics of wood formation compared to the control. The hypothesis that N addition stimulates wood formation in mature Chinese sweetgum was therefore rejected. The results showed similar wood formation dynamics with canopy and understory N addition. Consequently, the question whether canopy N addition is more effective than understory N addition in stimulating wood formation remained unanswered.

Neither canopy nor understory N addition increased intra-annual wood formation in our experiment. Our findings confirmed results from a previous experiment conducted in the same region where a 3-year understory N addition did not change xylem formation of *Pinus massoniana* Lamb. (Zhang et al., 2017). Lupi et al. (2012) also found that after 3 years of canopy N addition, wood production in mature *Picea mariana* Mill. was not affected by the treatment. These results might be attributed to a number of reasons that we describe and discuss in the following sections.

### Amount of N Addition

The amount of N addition and the difference between it and the ambient natural N deposition may play an important role in affecting tree growth. For instance, application of 0.35–0.5 kg N ha^-1^ year^-1^ (0.5 times natural deposition) did not increase *Picea mariana* wood production in Canada (Lupi et al., 2012). The similar result was also found in *Abies balsamea* Mill. after 1.1–2.7 kg N ha^-1^ year^-1^ addition, which corresponding to 0.2–0.5 times the natural N deposition (D’Orangeville et al., 2013). However, Zhao and Liu (2009) found that application of 250 kg N ha^-1^ year^-1^ (6.25 times the ambient N deposition) significantly increased biomass accumulation of *Picea asperata* and *Pinus tabulaeformis* in Central China. Similarly, tree height and radial growth of *Cryptocarya concinna* showed significantly positive response to 150 kg N ha^-1^ year^-1^ addition (4 times the natural deposition), but a negative response to 300 kg N ha^-1^ year^-1^ addition (8 times the natural deposition) in a subtropical forest of Southern China (Mo et al., 2008). Thus, the imbalance between the amount of natural and added N could play an important role in the effect of the treatment on trees. Moreover, like other environmental factors such as temperature and precipitation (D’Arrigo et al., 2004; Rossi et al., 2007; Sarris et al., 2007), a threshold of deposited N for tree growth may also occur (Mo et al., 2008).

Another possible explanation for the lack of effects of treatments might be that the experimental forest was already N saturated. In the same experimental site, Shi et al. (2016) found that the contents of soil exchangeable cations did not change in plots of 50 kg N ha^-1^ year^-1^ understory and canopy addition compared to control plots, and then raised a supposition that the forest canopy retention capacity might already be saturated by a high natural rate of N deposition (19.6 kg N ha^-1^ year^-1^). Under this scenario, most of the N added with the treatments would not be absorbed by the canopy and would be flushed away by the rain.

### Duration of N Addition

The duration of N addition is also another important factor for the effect of N deposition on tree growth. For example, 10 years of N addition (30 kg N ha^-1^ year^-1^) significantly increased woody biomass in four Northern hardwood forests in the United States (Pregitzer et al., 2008). Similarly, in a 30-year long N addition (34 kg N ha^-1^ year^-1^) experiment, 100 m^3^ ha^-1^ in wood-stem volume in the N treated plots exceeding the control plots was also reported in Sweden (Hogberg et al., 2006). However, after 3 years of N application (9–57 kg N ha^-1^ year^-1^), basal area increment and annual growth rate (after treatment) of *Abies balsamea* and *Picea mariana* were similar between control and treated plots in a boreal forest in Canada (Houle and Moore, 2008). Similarly, 3 years of N addition (25 kg N ha^-1^ year^-1^) did not stimulate wood formation of *Pinus massoniana* in the same experimental site as this study in Central China. Based on the previous studies, therefore, we concluded that the duration of N addition is thus another important factor affecting tree growth. Besides, since mature trees are less sensitive to nitrogen uptake than younger trees, non-woody plants and microorganisms, changes in nutrient cycling and its potential significant impacts on tree growth may need a longer duration to appear (Day et al., 2001; Vieira et al., 2008; Nasholm et al., 2009).

### N Is Not Main Factor In Affecting Radial Growth

Many environmental factors (temperature, precipitation, available nitrogen ect.) could affect radial growth (Mo et al., 2008; Huang et al., 2010; Rossi et al., 2016). The lack effect of N addition on radial growth may because that N is not the limiting factoring for radial growth in this experimental site. Evidences from the dendrochronology suggested that the radial growth in Qingling mountain (Central China) where near our experimental site was mainly affected temperature and precipitation (Hughes et al., 1994; Dang et al., 2007; Liu et al., 2009; Zhang et al., 2012). They found that, at low and middle elevations, the temperature in early spring and summer positively correlated with radial growth of *Abies fargesii*. Moreover, summer precipitation was correlated with tree growth at high elevations (Dang et al., 2007). Temperature and precipitation are also generally considered to be the main drivers of tree growth in other forests in Central China (Fang et al., 2012; Zhang et al., 2012; Zheng et al., 2012). For instance, tree ring-width of *Pinus tabulaeformis* exhibited positive and negative correlations with precipitation and temperature, respectively, in the Xiaolong Mountain area of Central China (Fang et al., 2012). In the Dabie mountains of Central China, radial growth of *Pinus taiwanensis* was positively correlated with spring temperature (Zheng et al., 2012). Therefore, we supposed that N may not be the main influencing factor for radial growth in the warm-temperate forest of Central China.

### Evidence from Chemistry

To the best of our knowledge, this is the first study that compared the effects of understory and canopy N addition on tree growth. Based on previous literature, larger leaves supplied with higher availability of N could increase photosynthesis, resulting in enhanced growth (Reich et al., 1998; Boussadia et al., 2010). In our experiment, both leaf size and N content in leaves were not affected by N addition, which was also consistent with the lack of result on the radial growth after N addition. Moreover, the study site is affected by Asian summer monsoons. When trees are growing, rain events are frequent (109 rainy days in April–October) and abundant, with 83% of the total annual precipitation (1,119 mm) falling during April–October^2^. The frequent and abundant rainy events may have flushed away the applied N, which may be proved by the lack change of N concentration (except for NH_4_^+^ in understory plots), pH (**Table 1**), and the percentage of cation exchange capacity detected after addition of 50 kg N ha^-1^ year^-1^ (Shi et al., 2016). Besides, in the soil, NH_4_^+^ is easily nitrified into NO_3_^-^ which is hardly captured and held by soil and readily lost through denitrification (Amberger, 1993). All these facts may indicate that, in our experimental site, 2.5 times the ambient N deposition is not able to change growing conditions, and consequently the wood formation of Chinese sweetgum, in the short term.

## Conclusion

In this study, either canopy or understory N addition (50 kg N ha^-1^ year^-1^) was applied to Chinese sweetgum for 3 years in a natural forest of Central China to simulate the impact of future increasing N deposition on tree growth. Contrary to our hypothesis, no effect of N treatments was observed on the growth and dynamics of wood formation. Similar growth dynamics were observed between canopy and understory N addition. Therefore, the question whether canopy N addition is more efficient than understory N addition in stimulating tree growth could not be answered by our experiment. The lack of effects on tree growth was probably due to the small amount or short duration of the N addition. The lower sensitivity of mature trees to environmental changes could also partially explain the lack of effects of the N treatments. The findings of the study suggest that 50 kg N ha^-1^ year^-1^, corresponding to 2.5 times the natural N deposition in Central China, is not able to significantly influence wood formation in the warm-temperate forest in the short term. The effects of N on tree growth observed by previous studies might be related to the duration of the experiment or the imbalance between the amount of natural deposition and the N added during treatments.

## Author Contributions

SZ did the main experiments, obtained the whole dataset and wrote the whole manuscript. SR helped the first author with the data analysis and gave many constructive suggestions for the analysis and writing work. J-GH the first author’s director, funded the first author to conduct this study and gave many constructive suggestions for the writing work. SJ and BY helped with the experiment and raised many good questions and solutions to the present work. WZ and QY provided some useful and supportive data and gave many good advices to this work.

## Conflict of Interest Statement

The authors declare that the research was conducted in the absence of any commercial or financial relationships that could be construed as a potential conflict of interest.

## Footnotes

1 http://data.cma.cn

2 http://data.cma.cn
